# BlueEdge neural network approach and its application to automated data type classification in mobile edge computing

**DOI:** 10.1038/s41598-025-30445-z

**Published:** 2025-12-12

**Authors:** Nagwa Elmobark, Haitham El-ghareeb, Sara Elhishi

**Affiliations:** Department of Computer Science, University of Mansoura, Mansoura, Egypt

**Keywords:** Edge computing, Neural network classification, IoT data preprocessing, Automated data type detection, Resource-efficient computing, Computational science, Computer science, Information technology

## Abstract

Owing to the increasing number of IoT gadgets and the growth of big data, we are now facing massive amounts of diverse data that require proper preprocessing before they can be analyzed. Conventional methods involve sending data directly to the cloud, where it is cleaned and sorted, resulting in a more crowded network, increased latency, and a potential threat to users’ privacy. This paper presents an enhanced version of the BlueEdge framework—a neural network solution designed for the automated classification of data types on edge devices. We achieve this by utilizing a feed-forward neural network and optimized features to identify the presence of 14 distinct data types. Because of this, input data can be preprocessed near its source, and not in the cloud. We utilized a comprehensive dataset comprising 1400 samples, encompassing various data formats from around the world. Compared with rule-based methods, experimental assessment achieves better performance, and results in reduced data transmission (reduced by 62%) and processing latency (78 times faster than cloud-based systems), with resource efficiency comparable to low-end mobile devices. Additionally, our strategy demonstrates strong performance under various data conditions, achieving accuracy levels of over 85% on datasets that may include variations and a noise level as high as 20%. The approach used here is capable of processing data for IoT devices used in education, which can lead to more efficient connections with the cloud and better privacy preservation.

**Supplementary Information:**

The online version contains supplementary material available at 10.1038/s41598-025-30445-z.

## Introduction

 The exponential growth of Internet of Things (IoT) devices has led to an unprecedented surge in data generation in the network. According to current estimates, by 2025, over 75 billion IoT devices will be connected to the internet, generating more than 79.4 zettabytes of data^1^. This massive volume of heterogeneous data presents significant challenges for traditional cloud-centric processing paradigms, particularly in terms of network bandwidth consumption, processing latency, and privacy concerns.

Traditional data processing techniques typically involve transmitting raw data from edge devices to centralized cloud servers, where data cleaning, classification, and analysis are performed^2^. This centralized model, while computationally efficient, introduces several inefficiencies. First, transmitting unprocessed data consumes valuable bandwidth, contributing to network congestion. Second, the round-trip delay between data generation and the result retrieval can be prohibitively high for time-sensitive applications. Third, sending sensitive data to external servers raises significant concerns regarding privacy and security^3^.

Mobile Edge Computing (MEC) has emerged as a promising paradigm to address these challenges by bringing computational capabilities closer to data sources^4^. MEC can significantly reduce bandwidth consumption and latency by processing data locally or at nearby edge servers while improving data privacy. However, effective edge processing requires intelligent systems capable of understanding and classifying data types with minimal computational resources, a capability that remains underdeveloped in current edge computing frameworks.

Our previous work introduced BlueEdge, a fog-edge mobile application that shifts data cleaning responsibilities from cloud servers to edge devices^5^. BlueEdge significantly improved resource utilization and processing time compared to traditional data warehouse tools. However, its initial implementation relied on predefined rules and patterns for data type identification, which limited its adaptability to diverse and evolving data formats.

This paper extends the BlueEdge framework by introducing a neural network-based approach for automatic data type classification on resource-constrained mobile devices. Our approach leverages carefully engineered feature extraction techniques and a lightweight neural network architecture to identify 14 data types commonly encountered in mobile and IoT applications. This intelligent classification enables more robust data preprocessing directly at the edge, reducing the volume of data transmitted to cloud servers and enhancing overall system efficiency.

The primary contributions of this paper are:

1. A lightweight neural network architecture designed specifically for data type classification on resource-restricted mobile edge devices.
2. A comprehensive feature extraction methodology that captures distinctive characteristics of various data types while maintaining computational efficiency.
3. A diverse training dataset encompassing 14 data types with global variations and format diversity.
4. Extensive evaluation demonstrating the system’s classification accuracy, resource consumption, and latency reduction compared to cloud-based alternatives.
5. Implementation insights for integrating neural network-based classification within mobile edge computing environments.

As far as we understand, the majority of previous literature on edge data processing is dedicated to either rule-based heuristics or larger models that are not easily portable to resource-limited devices. This paper demonstrates that with a carefully designed set of features and a lightweight three-layer neural network, one can achieve high classification accuracy and satisfy the strict resource limitations inherent in mobile edge computing environments. In-depth performance indicators are provided in “Results”. Another addition is that the model has been integrated into the existing BlueEdge framework, which enhances its cleaning and processing capabilities at the device level. 

## Literature review

### Data cleaning and classification in cloud environments

For a long time, people have used cloud-based data warehouses to perform data cleaning and classification, as they offer significant processing power. Based on their research in^6^, Côté et al. laid the groundwork for the modern data preprocessing methods commonly applied on servers. Our prior framework integrated statistical and machine learning approaches to enhance data cleaning performance, providing a basis for automated data type detection^7^.

Raman and Hellerstein^8^, as well as Hulsebos et al.^9^, are among those who have looked into automatic data type detection. A recent paper by Ali et al.^10^ improved upon previous work by utilizing transformers to label data across various topics, achieving outstanding results. In their study, Albshaier et al.^11^ developed federated learning models for evaluating multichannel data quality and addressed privacy challenges during cloud-based data processing. Although they are accurate, these systems are best suited for servers and require powerful processors that today’s edge devices cannot provide, so alternative options are necessary.

### Edge computing for data processing

Bringing computational power near data is what edge computing promises to address challenges^12^. Cloudlets, developed by Kanagarla^13^, were set as standards for Mobile Edge Computing by the European Telecommunications Standards Institute (ETSI)^14^. The latest developments have primarily focused on innovative edge management and effective resource allocation^15^.

Researchers in edge computing today are paying more attention to preprocessing with AI. Poncinelli Filho et al.^16^ introduced specialized machine learning frameworks for edge computing, and Golpayegani et al.^17^ proposed methods to adapt edge computing for data from the Internet of Things (IoT). According to^18^, Shi et al. developed unique edge-cloud collaboration strategies that enhance data processing based on real-time network conditions. Our comparative study on machine learning and deep learning trade-offs^19,20^ further supports the choice of lightweight neural networks in edge environments. Due to this gap, it is crucial to develop methods suitable for identifying different data types on mobile devices.

### Machine learning for resource-constrained devices

Running machine learning models on low-powered devices is difficult and requires specific optimization measures. The authors in^21^ developed MobileNets, which are efficient convolutional neural networks designed for mobile vision. In^22^, Zhang et al. introduced ShuffleNet, specifically designed for use on mobile devices. Recent improvements have focused on developing lightweight, efficient methods for mobile use and attention models that can operate with limited resources^23,24^. The vast majority of these architectures, however, are vision-oriented, such as MobileNet and ShuffleNet, and generally require a model size of a few megabytes. By contrast, BlueEdge is specifically a small, compact (5KB) feed-forward neural network designed for use in classifying structured data types on resource-constrained devices. Recent developments in model compression now enable the use of artificial neural networks with reduced memory requirements and increased efficiency on mobile devices, as reported by Han et al.^25^ and other studies^26^. Several new methods for pruning and knowledge distillation have been developed, particularly for edge applications, as a result of recent research^27,28^. Using only integer operations is now possible, thanks to the work by Jacob et al.^29^, who also proposed adaptive quantization techniques^30^. Still, despite this progress, most research has not explored using neural networks to classify various data types in edge scenarios, as existing options tend to either utilize strong cloud resources or match specific applications, rather than covering multiple kinds of data.

### Recent advances in edge AI and mobile neural networks (2024–2025)

Edge AI trends have enabled experts to build neural networks that can be deployed on simple devices, addressing the computational issue that affects mobile edge applications. Fujiwara and Kawahara^31^ proposed new training algorithms for binarized neural networks (BNNs) that utilize ternary gradients, specifically designed for IoT devices employing magnetic RAM-based compute-in-memory systems. It demonstrates that advanced neural networks can be used effectively with limited resources, which are crucial for edge computing.

Over time, quantization techniques have improved significantly, as lower bit sizes can maintain accuracy close to the reference and require only a fraction of the usual computational resources^32,33^. Singh et al. presented edge-based data space methods to integrate data through distributed technology for real-time processing^34^. However, Pacheco et al.^35^ also developed multi-armed bandit techniques for early-exit neural networks, aiming to save time by exiting when results exceed a threshold.

Improvements in neural architecture for mobile devices have been driven by new compression and pruning techniques designed for use in resource-constrained environments. Asymmetric exponent techniques were studied by Pietrołaj and Blok^36^ to bring significant improvements in parameter efficiency; the authors also investigated new approaches for controlling floating-point variables and representations, especially in devices with limited resources. Thanks to these optimizations, intelligent applications can operate efficiently on edge devices with limited computing power, making it easier for our BlueEdge framework to deliver its intended solution.

### Hybrid edge-cloud architectures and recent developments

It has become clear to researchers that using either edge or cloud computing alone is insufficient. Hence, they have proposed combining the best aspects of both approaches. In^37^, Ur Rehman and colleagues introduced RedEdge, enabling mobile edge devices to perform most of the data mining with minimal data transmission to the cloud. Nevertheless, they focus on reducing data size through compression rather than classifying it at an early stage.

In recent times, scholars have paid more attention to how workloads are distributed and how tasks are properly organized^38^. Heidari et al. developed federated learning for edge computing to enhance the security of IoT-enabled devices, and Shaheen et al. proposed adaptive federated learning frameworks designed for resource-constrained IoT devices, leveraging edge intelligence and clustering. The latest research by Guo et al.^39,40^ presents green federated learning methods that help preserve performance while reducing energy consumption in separate edge systems.

Previously, we worked on BlueEdge^5^ and found that performing data cleaning on edge devices is possible, resulting in faster processing and reduced resource utilization compared to traditional data warehouses. Still, its rules for different kinds of data made it difficult to handle all data formats effectively.

### Research gap and contribution

Research indicates a significant gap in understanding how to classify data types for edge computing. Although considerable effort has been invested in data cleaning for clouds and enhancing machine learning on mobile devices, the combination of these efforts with neural networks for edge classification has not been widely explored.

Although the accuracy is high with today’s cloud-based solutions, using them requires more computing than edge devices can offer. Most existing work on mobile machine learning focuses on computer vision and general inference rather than structured data classification. Experts in edge computing primarily focus on managing resources and transferring workloads to other devices; however, they are still limited in their ability to effectively interpret data. Edge AI technology has recently improved, but its application in automated data classification remains to be explored.

We have developed a lightweight solution that utilizes neural networks and is particularly well-suited for data type classification on systems with limited resources. Thanks to our method, raw data does not need to be transferred to servers, which helps improve both the performance and privacy of the system while utilizing the latest mobile neural network methods.

## Methodology

### Research design overview

This study follows a design science paradigm^41^, which is well-suited for developing and evaluating novel technological artifacts. Our approach involves four phases: (1) problem identification and motivation, (2) solution design, (3) development and implementation, and (4) evaluation and validation. This structured approach enables us to systematically address the challenges of data type classification in edge computing environments, ensuring both scientific rigor and reproducibility.

This paradigm consists of four phases, which are described in more detail in Supplementary Section S1.

### Data collection and sampling

The sample was stratified into 14 different types, and the full dataset specification is provided in “Dataset description and preparation” and in Supplementary Table S1.

### Experimental design

We designed our experiments using a comparative evaluation framework^42^, systematically comparing our proposed neural network approach against established baselines. Our comparative evaluation framework is similar to the use of metrics, controls, and statistical procedures that are outlined in “Experimental objectives and design” and “Evaluation metrics and procedures” (Supplementary Section S3). for neural network initialization to ensure reproducible results, standardized hardware configurations across all comparative experiments, controlled environmental conditions to provide accurate measurements of resource consumption, and cross-validation techniques to minimize overfitting and sampling bias.

### System development methodology

The development process was based on creating a minimal viable prototype and a final mobile-ready prototype; the details of the implementation will be presented in “Neural network implementation”.

### Evaluation framework

Our evaluation framework combined quantitative performance metrics with qualitative insights from real-world deployment. The primary quantitative evaluation employed stratified k-fold cross-validation (k = 10)^43^ to assess classification performance across multiple data partitions, thereby reducing the impact of sampling variations on the reported results.

For baseline comparisons, we implemented three established approaches: rule-based classification, using regular expressions and heuristics, cloud-based deep learning (Sherlock 34), and commercial data cleaning tools (WinPure Clean). Each approach was evaluated under identical conditions using the same datasets and testing environments. The statistical significance of performance differences was assessed using McNemar’s test^44^, which is particularly appropriate for paired nominal data in classification tasks.

Additionally, we performed ablation studies to quantify the contribution of individual components to overall system performance. These studies systematically eliminated or modified specific features, architectural elements, or optimization strategies to measure their impact on classification accuracy and resource consumption.

### Implementation and deployment

The implementation phase translated our theoretical design into a functional prototype suitable for pilot deployment. We developed the system using TensorFlow Lite for the neural network components and the Kivy framework for the mobile user interface. This technology stack was chosen based on its optimization capabilities for mobile environments and cross-platform compatibility.

We used TFLite inference and a cross-platform deployment based on Kivy UI; the implementation steps are described in “Neural network implementation” and Supplementary Section S10.

### Ethical considerations

While our research primarily focuses on the technical aspects of data processing, we have also recognized and addressed several ethical considerations. First, all data used for training and testing were either synthetically generated or properly anonymized to protect privacy. Second, we considered the potential power impact of edge processing and designed our system to minimize battery consumption. Finally, we ensured that our preprocessing approach preserved data integrity while enhancing privacy by reducing data exposure.

Before experimentation, all personally identifiable information (PII) in the university dataset was anonymized. Format-preserving techniques were used to implement anonymization, ensuring that privacy was preserved without distorting the statistical distribution or structure of the data. In this way, the semantic integrity necessary for providing a meaningful evaluation was preserved.

## 4 BlueEdge auto data type classification framework

### 4.1 System architecture overview

**Fig. 1:**
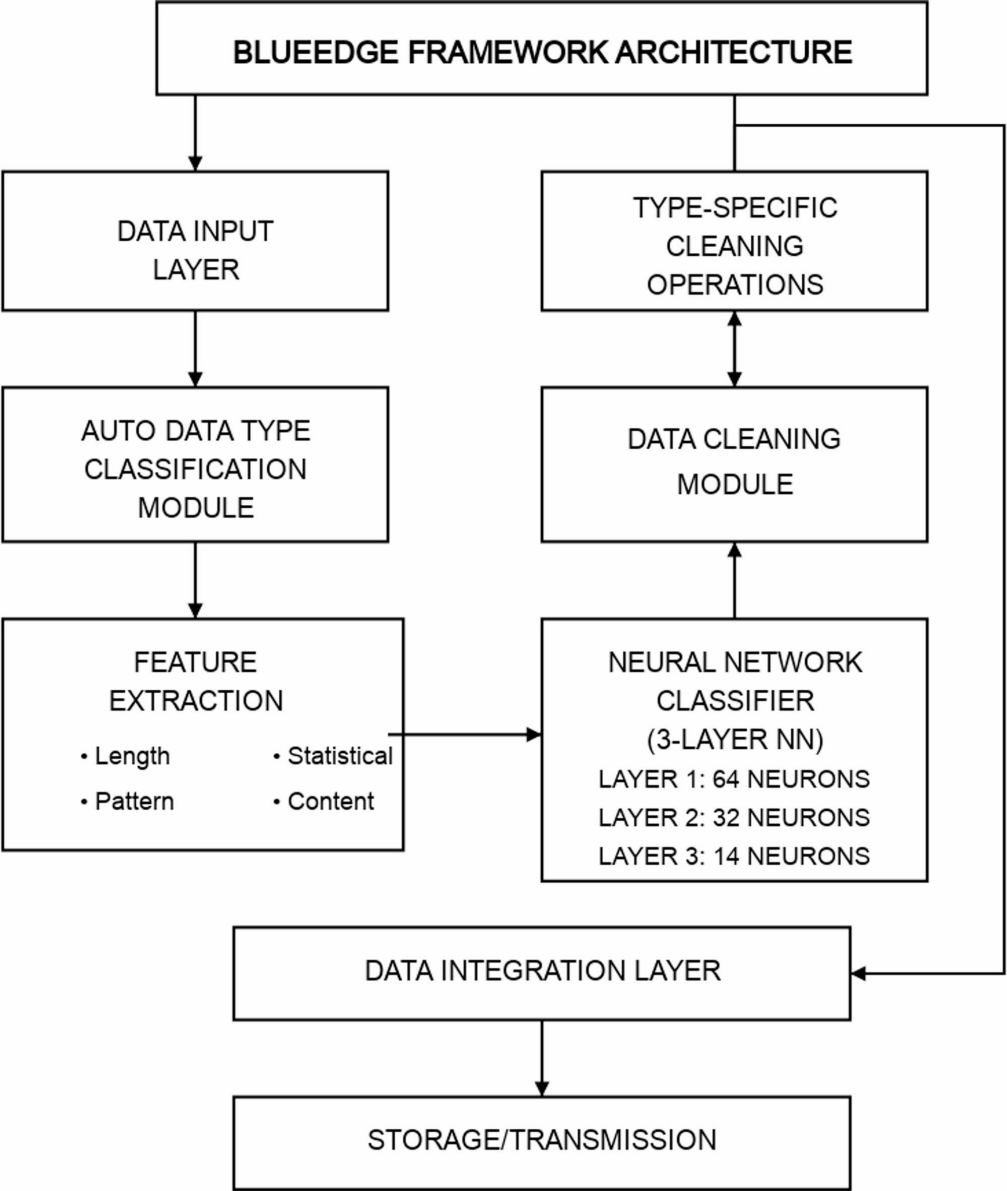
Architecture of the BlueEdge auto data type classification framework.

The BlueEdge Auto Data Type Classification framework extends our previous BlueEdge architecture^5^ by incorporating an intelligent neural network-based classification component that operates entirely at the mobile level. Figure 1 illustrates the overall architecture of the enhanced system.

The framework includes four primary components: (1) the Data Input Layer, which handles incoming data from various sources; (2) the Auto Data Type Classification Module, which employs our neural network model to identify data types; (3) the Data Cleaning Module, which applies appropriate cleaning techniques based on the identified types; and (4) the Data Integration Layer, which prepares the processed data for storage or transmission.

This modular design allows efficient data processing directly on edge devices while maintaining low resource consumption. The key innovation lies in the Auto Data Type Classification Module, which replaces the rule-based approach of the original BlueEdge system with a more flexible and robust neural network solution.

### Neural network design considerations for mobile edge

Designing a neural network for deployment on resource-constrained mobile devices offers unique challenges. Unlike cloud-based systems that can leverage deep, complex architectures, mobile edge solutions must carefully balance accuracy with computational and memory efficiency. Our approach prioritizes lightweight design while maintaining robust classification capabilities.

We evaluated various neural network architectures, including convolutional neural networks (CNNs) and recurrent neural networks (RNNs), before selecting a feed-forward structure with carefully tuned parameters. While CNNs excel at spatial pattern recognition and RNNs at sequential data processing, our experiments showed that a well-designed feed-forward network with appropriate feature engineering achieves comparable accuracy with significantly lower computational requirements.

We prioritize a lightweight feed-forward design over CNN/RNN for on-device efficiency; final architecture and sizes are described in “Model architecture design”.

### Feature extraction methodology

An essential aspect of our approach is the feature extraction methodology, which transforms raw data into a structured representation suitable for neural network processing. Rather than using raw inputs, which would require a more complex network structure, we extract meaningful features that capture the essential characteristics of different data types.

Given the 40 features, the theoretical considerations informed them to make sure that the data is covered in four complementary dimensions, including structural (e.g., length, number of digits), statistical (e.g., entropy, character proportions), pattern-based (e.g., regular expressions representing separators), and semantic indicators (e.g., domain-specific tokens). This multidimensional design is based on the hypothesis that the useful classification of types needs signals of orthogonal categories of features, and not based on one component of the data.

Our feature extraction pipeline consists of four complementary components:

#### Length features

Length features capture the data’s basic structural characteristics, including total character count, word count, and various special characters. These features are simple yet informative.

These features provide baseline discrimination between data types with distinct length profiles (e.g., short binary values versus longer text descriptions). We extract six length-based features as shown in Table 1.

**Table 1:** Length-based feature extraction and pattern classification.

Feature	Description
Total character length	The total number of characters in the input data.
Word count	The number of words in the input data (if relevant).
Digit count	The number of digits present in the data.
Alphabet character count	The count of alphabetic characters within the input.
Space count	The number of spaces in the data.
Special character count	The number of special characters (punctuation, symbols, etc.).

#### Pattern features

Pattern features identify unique structural patterns through regular expression matching. For each data type, we define characteristic patterns and compute Boolean features indicating their presence. We extract 12 pattern-based functions for the usage of regular expressions designed to identify them, as shown in Table 2.

**Table 2:** Overview of pattern-based features and data pattern types.

Pattern Type	Description
Email format	Pattern for email addresses (e.g.,username@domain.extension).
Phone number formats	Various regional phone number formats.
Date formats	Differentformatsfordates(e.g., MM/DD/YYYY, YYYY-MM-DD.
SSN pattern	Pattern for Social Security Numbers (e.g., XXX-XX-XXXX).
URL structure	URL format, including protocol (e.g., protocol://domain/path).
Numeric ID patterns	ID numbers are composed entirely of digits.
Currency patterns	Patterns that combine currency symbols with numbers.
Percentage patterns	Patterns for percentages (numbers followed by the % symbol).
Binary patterns	Patterns indicating binary values (e.g., true/false, yes/no).
Name-like patterns	Patterns resembling names, usually with capitalized words.
Geographic coordinate patterns	Patterns for geographical coordinates (latitude/longitude).

#### Statistical features

Statistical features capture the distribution of characters within the data. These features help distinguish between data types with similar structures. We extracted seven statistical features as shown in Table 3.

**Table 3:** Character-based ratios and entropy measures for data analysis.

Feature	Description
Ratio of digits to total length	The percentage of digits compared to the total character length.
Ratio of alphabetic characters total length	The percentage of alphabetic characters compared to the total length.
Ratio of uppercase to lowercase characters	The ratio of uppercase letters to lowercase letters in the data.
Ratio of special characters to total length	The percentage of special characters (e.g., punctuation, symbols) of the full length.
Ratio of whitespace to total length	The percentage of whitespace characters compared to the entire character length.
Count of unique characters to total length ratio	The ratio of unique characters in the input compared to the total length.
Shannon entropy of character distribution	A measure of uncertainty or randomness in The distribution of characters.

#### Content features

Content features examine the semantic context of the data by identifying domain-specific keywords and patterns. These features help distinguish between data types that may have similar structural properties but different semantic contexts. We extracted 15 content-based features, as shown in Table 4.

**Table 4:** Hardware configuration specifications.

Device category	Device model	Processor	RAM	Operating system
Low-end mobile	Samsung Galaxy A10	Exynos 7884	2GB	Android 9.0+
Mid-range mobile	Google Pixel 4a	Snapdragon 730G	6GB	Android 11+
High-end mobile	Samsung Galaxy S21	Exynos 2100	8GB	Android 11+
Cloud server	Virtual machine	Intel Xeon E5-2680 v4 (8 vCPUs)	32GB	Ubuntu 20.04 LTS

These 40 features comprehensively represent the input data, enabling our neural network to distinguish among distinct data types despite restricted computational resources.

### Classification approach

Our classification approach combines the extracted features with a neural network model to predict the most likely data type for a given input. The overall classification process involves three main steps:

1. Feature Extraction: The raw input data undergoes the feature extraction process described in “Feature extraction methodology”, resulting in a 40-dimensional feature vector.
2. Neural Network Inference: The feature vector is fed into the trained neural network, which produces a probability distribution across the 14 possible data types.
3. Decision Making: The system selects the data type with the highest probability as the classification result, provided the probability exceeds a configurable confidence threshold (default: 0.6).

To enhance reliability, we incorporate a confidence threshold mechanism. The system flags the sample for potential manual verification if the highest probability falls below the threshold. This approach prevents the misclassification of ambiguous inputs while maintaining high automation rates for identifiable data types.

weighted arbitration. In practice, several sets of features can be fired simultaneously “patterns ‘phone-number’ and ‘numeric-ID’ “. BlueEdge resolves this conflict by weighting all 40 features of the neural network. Through training, the network learns to maximize the role of structural, pattern, statistical, and content cues together, such that corroborated evidence is boosted at the expense of spurious matches. Instead of using the hard rule of precedence, the decision is made based on the softmax posterior of all classes. Also, we use a confidence threshold of 0.6: any prediction with a top percentage class probability equal to or less than it is marked as needing manual confirmation instead of being assigned. This algorithmically addresses overlapping sets of features and minimises misclassification of ambiguous examples.

Conflicting cases are dealt with using a hybrid approach: the neural network generates a softmax probability distribution over all types of data. When the maximum probability falls below the 0.6 confidence threshold, the sample is not categorized to a class, but it is marked to be reviewed manually. This backup system minimizes systematic errors that arise when case features overlap.

### Cleaning strategy selection

Once the data type is identified, the system selects appropriate cleaning strategies based on a predefined mapping between data types and cleaning operations (see Supplementary Table S1). Type-specific cleaning is selected via a predefined mapping (Supplementary Table S1); trade-offs with deeper models are discussed in “Ablation study results”.

The reason why the three-layer feed-forward architecture was chosen was that it represents a compromise between representational capacity and resource efficiency. Deeper architectures yielded small accuracy improvements (Table S4), but at a significantly higher computational and memory cost. The selected configuration, therefore, is a theoretically based trade-off between precision and deployability of low-resource edge devices.

### Integration with existing BlueEdge framework

**Fig. 2:**
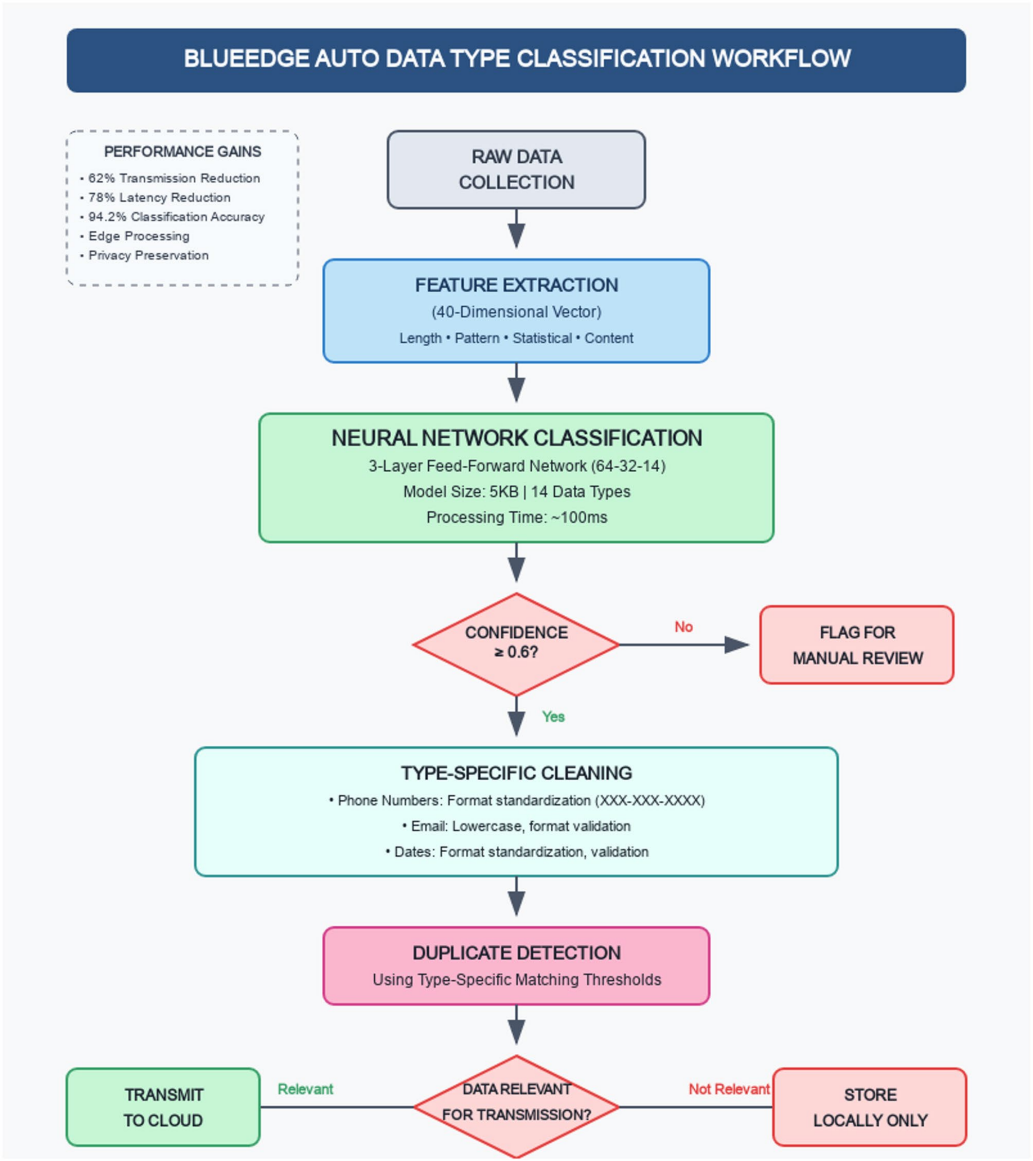
Integration workflow of auto data type classification with BlueEdge framework.

The Auto Data Type Classification module is designed to integrate seamlessly with the existing BlueEdge framework, enhancing its data cleaning capabilities without compromising its performance. Figure 2 illustrates the integration workflow.

In the enhanced workflow, the neural network-based classification occurs early in the processing pipeline after data collection. The classification results are then used to guide subsequent cleaning operations, ensuring that each data object receives appropriate type-specific processing.

The key advantage of this integrated approach is that it preserves the efficiency benefits of the original BlueEdge framework while adding the flexibility of neural network-based classification. By performing intelligent processing at the edge, the system can make informed decisions about data cleaning and data transmission, reducing the amount of data sent to cloud servers.

## Experimental and methodology

### Experimental objectives and design

We tested our method against five key objectives, including measuring the system’s accuracy with various types of data, the resources it consumes on smartphones, the time it takes to operate compared to the cloud, the amount of data transmitted, and its resilience against real-world variations. To ensure the internal validity of our experiments, we employed several methods, including the use of fixed random seeds for initializing neural networks, consistent configurations across all hardware devices, controlled room parameters to facilitate precise resource allocation, and cross-validation to minimize overfitting and increase the likelihood of finding more robust samples. The experiments evaluated the system’s performance by measuring accuracy, precision, recall, and F1-score. Resource usage was monitored by tracking memory usage, processing time, and power consumption. The tests also monitored how fast and efficiently data was transferred, along with checking how long it took and how much bandwidth was used.

### Hardware and software configuration

We performed experiments on three devices to gain a comprehensive understanding: a low-end Samsung Galaxy A10 (Exynos 7884, 2 GB RAM), a mid-range Google Pixel 4a (Snapdragon 730G, 6 GB RAM), and a high-end Samsung Galaxy S21 (Exynos 2100, 8 GB RAM). Table 4 presents the complete hardware features of these devices, along with the virtual cloud server used to evaluate their performance. We conducted our comparisons using a virtual machine with 8 vCPUs, 32 GB of RAM, and Ubuntu 20.04 LTS to demonstrate a typical cloud server configuration. We tested the apps under various network environments, including high-speed Wi-Fi, 4G LTE, and 3G. TensorFlow Lite was chosen to optimize neural network inference on mobile devices. Kivy was used to provide a consistent interface across devices. Python 3.8 + was the primary programming language, and Firebase enabled real-time data synchronization.

### Dataset description and preparation

We utilized three different datasets to provide a detailed review of the system’s efficiency and usefulness in mobile-edge computing settings. We used a dataset comprising 1400 instances, with each data type having 100 samples for every category. A stratified sampling method was employed to ensure that every aspect of formatting was represented equally in the dataset. A set of 210 samples was reserved for the test phase to ensure that all the data remained balanced. A total of 3615 real-world university registration records were used for this project, which included students’ names, ID numbers, and email addresses. A subset of 500 records was set aside to verify and confirm the results. Additionally, we created a special dataset of 200 samples for testing challenging cases, which included unusual formats, various national dataset variations, unclear items, and common writing errors to assess the system’s reliability and its ability to generalize. The datasets and their distribution are presented in Table 5.

**Table 5:** Dataset characteristics and distribution.

Dataset type	Total records	Data types covered	Purpose	Validation method
Primary training	1,400 samples	14 distinct types (100 each)	Model training and validation	Stratified 10-fold CV
University registration	3,615 records	Names, IDs, emails	Real-world testing	500 manually labeled
Synthetic challenge	200 samples	Edge cases and variations	Robustness evaluation	Expert annotation

The initial dataset of 1400 samples (100 samples of each type) is quite small, but it was used to provide a controlled benchmark of features first. Training and validation of the model were performed on a larger, anonymized dataset from the university (3615 records), and then systematically evaluated using 10-fold stratified cross-validation. The guaranteed accuracy of the report was achieved without compromising the small sample size, and generalization was preserved.

In the case of the university data (3,615 records), the personally identifiable information (PII) was anonymized. Format-preserving techniques were employed to anonymize the data without altering its structural and semantic elements. In particular, first-middle-last name combinations have been replaced with synthetically valid but meaningless first-middle-last name combinations, email addresses have been substituted by generic email addresses (e.g., userXXX@gmail.com), phone numbers and the ID numbers have been substituted with randomly generated but equally lengthy and formatted digit strings (including separators where applicable). Birth and registration dates were adjusted to a similar reasonable range. These ensured that complete privacy was achieved without compromising the semantic integrity and statistical diversity required for experimental feature extraction and classification.

All of the datasets were preprocessed in a planned manner, involving the creation of a 40-dimensional feature vector that encompasses several aspects, including length, pattern, statistics, and content. After that, the data were processed using a standardized normalization pipeline, and all labels were verified for accuracy before applying proper anonymization methods to ensure privacy. There are 14 specific data type categories, and each requires a particular preprocessing strategy, as explained in the detailed mapping framework provided.

#### Cross-domain validation datasets

It has been proven that the BlueEdge neural network performs best on the primary educational dataset made available from universities. Such a detailed validation explains how the model performs in the area of academic intelligence.

Additional future steps will confirm the performance by using healthcare records, financial information, and IoT data. Such expansions are designed to showcase the versatility of our approach for various applications, thereby enhancing the reliability and feature richness of the BlueEdge framework.

### Baseline comparison methods

For an overall evaluation, we compared our method with four well-known methods from different fields in machine learning. According to our first BlueEdge method, all classification was performed using predefined rules on patterns that included regular expressions, with the assistance of NLTK, along with Levenshtein distance measurements and a similarity threshold of 0.25. Cloud Sherlock offered an advanced semantic class system that ran in the cloud, utilizing a powerful deep neural network engine for comprehensive resource support. The system was evaluated in its original form in the cloud, as well as a modified version directly on a device, to obtain accurate results. WinPure Clean was our primary commercial data cleaning tool, representing strong enterprise solutions and regular server configurations designed to identify duplicates and ensure data consistency. To top it off, our RegEx Classifier baseline carried out effective and efficient processing by using hand-designed regular expressions for every type of data. All the critical features of each baseline model are described in Table 6.

**Table 6:** Baseline method specifications.

Method	Implementation	Deployment	Key features	Optimization focus
BlueEdge neural network	Lightweight 3-layer NN	Mobile edge	40-dim features, 5KB model	Accuracy + efficiency
Rule-based classification	NLTK + RegEx patterns	Mobile edge	Levenshtein distance	Pattern matching
Cloud Sherlock	Deep neural network	Cloud server	Semantic type detection	Server-grade accuracy
WinPure clean	Commercial tool	Server	Enterprise data quality	Professional cleaning
RegEx classifier	Hand-crafted patterns	Mobile/Server	Pattern hierarchy	Traditional rules

#### Future work: comparison with state-of-the-art mobile AI models

For future research, we intend to utilize the latest mobile AI frameworks specifically designed for mobile and edge devices. adapt MobileNetV3 for type classification by removing the original output layer and tuning the input pipeline to support 40-dimensional feature vectors. Models based on EfficientNet-Lite will also be created for devices with low memory, by training them for use with quantization and making custom feature layers for tabular data.

We are working to analyze Quantized BERT architectures, as they provide an efficient way to run transformer models on mobile devices. We plan to make improvements by applying 8-bit quantization and knowledge distillation, as well as designing personalized tokenization systems and enhanced attention models tailored to our specific needs. FedAvg methods will also be studied, allowing us to compare the performance of centralized training systems with that of privacy-preserving distributed models in mobile applications.

This planned work will enable us to fairly compare BlueEdge with recent mobile AI techniques and identify which changes lead to noticeable improvements under everyday mobile-based realities.

### Evaluation metrics and procedures

We employed both numerical and experimental methods within our framework to obtain an accurate measure of the system’s capabilities (see Supplementary Table S3 for more details). We evaluated the performance of models by using standard measures for several classes, including the percentage of correct samples, precision, recall, F1-score, and confusion matrices for in-depth error analysis. Scores were compared for all data types and for individual ones to determine if there were any differences in their performance.

Android system reports was used to determine how much memory, CPU, and battery are utilized by edge devices, and memory handling was observed through callbacks. It was found that appending data, using the network, and decision-making were the primary steps, so opportunities for improvement were sought in each of these steps.

The system was evaluated by demonstrating reduced data transmission, saved bandwidth, and reduced overall latency, which highlighted the primary benefits of processing data at the edge. Results were measured through 10-fold stratified cross-validation, and McNemar’s test was performed to determine any significant changes in classification performance across various data groups. To test robustness, data were corrupted with noise up to 30%, and entire components were removed to assess the effect of each part. Every experiment was carried out ten times, and the confidence intervals were reported to ensure the results were reliable and could be repeated.

## Neural network implementation

### Model architecture design

Designing a neural network for deployment on resource-constrained mobile devices presents unique challenges that require careful balance between classification accuracy and computational efficiency. Unlike cloud-based systems that can leverage deep, complex architectures, mobile edge solutions must prioritize lightweight design while maintaining robust classification capabilities.

After evaluating various neural network architectures, including CNNs and RNNs, we selected a feed-forward structure with carefully optimized parameters. The final architecture consists of a three-layer neural network with 64 neurons in the first hidden layer, 32 neurons in the second hidden layer, and 14 output neurons corresponding to our target data types. We use ReLU activation functions in the hidden layers and softmax activation in the output layer, with dropout regularization (rate: 0.2) and L2 regularization (λ = 0.001) to prevent overfitting. This configuration achieves a model size of approximately 4.3 KB, well within our 5,000-byte target for edge deployment.

We systematically validated these architectural choices through comprehensive ablation studies that optimized trade-offs between classification accuracy and resource consumption. Table 7 presents the quantitative results of our optimization process across multiple design dimensions.

**Table 7:** Architecture ablation study results.

Configuration	Accuracy (%)	Model size (bytes)	Processing time (ms)	Memory usage (KB)	Power (mAh/1K)
Network depth					
1-layer (32 neurons)	78.3	1,200	45	2.8	1.8
2-layer (32 − 16)	89.7	2,800	68	3.2	2.1
3-layer (64-32-14)	94.2	4,300	87	5.0	2.8
4-layer (64-32-16-14)	94.5	6,950	123	7.1	3.4
Neuron count					
16-16-14	87.1	1,850	52	3.1	2.0
32-32-14	91.8	3,200	71	4.2	2.5
64-32-14	94.2	4,300	87	5.0	2.8
128-64-14	94.7	12,400	165	9.8	4.2
Activation functions					
ReLU	94.2	4,300	87	5.0	2.8
Tanh	92.8	4,300	103	5.0	3.1
Swish	94.9	4,300	142	5.0	3.7
Regularization (dropout)					
0.1 dropout	92.1	4,300	87	5.0	2.8
0.2 dropout	94.2	4,300	87	5.0	2.8
0.3 dropout	93.8	4,300	87	5.0	2.8
0.5 dropout	91.7	4,300	87	5.0	2.8

When we compared network depth, our chosen 3-layer model gave better results (accuracy of 94.2%) than other, simpler models. The representational capability of single-layer networks was insufficient (78.3%), and only a minor improvement was observed in the 4-layer networks (94.5%), which utilized more resources (6950 vs. 4300 bytes). The 64-32-14 configuration proved to be the best, achieving nearly the highest accuracy (94.9%) with fewer neurons, compared to the 128-64-14 configuration, where the accuracy increase was minimal, despite requiring more memory and running much longer.

By evaluating the activation function, it was clear that ReLU performed best on mobile, achieving approximately the same accuracy (94.2%) as Swish (94.9%), while requiring only 87% of the time (42 ms shorter inference time) than Swish. It was determined that with a 0.2 dropout rate, the model accurately generalized to every dataset and kept good cross-validation scores. These test results demonstrate that the chosen structure is the most suitable for mobile networks, as it strikes a balance between powerful features and efficiency.

### Training methodology

The training of our neural network was performed using supervised learning with the dataset described in “Experimental and methodology”. The training steps were intentionally organized to simplify the process.

The model is trained to learn class-discriminative weights through cross-entropy minimization with L2 regularization and dropout, thereby avoiding over-reliance on any single feature. The ablation in Table 8 also demonstrates how the network prioritizes pattern features to resolve conflicts (− 10.7% accuracy when ablated), and structural and content features are complementary, aiding in disambiguating borderline cases.

**Table 8:** Feature category contribution to classification accuracy.

Feature configuration	Accuracy	Change from full model
All features (complete model)	94.2%	—
Without length features	91.8%	-2.4%
Without pattern features	83.5%	-10.7%
Without statistical features	92.7%	-1.5%
Without content features	90.3%	-3.9%
Only pattern features	78.2%	-16.0%
Only statistical features	67.5%	-26.7%
Without feature normalization	92.9%	-1.3%

For optimal performance. We set the initial weights using the He initialization method^45^, which is known to be the most effective for networks with ReLU activations. Adam was chosen as the optimizer^46,47^ with learning rate, beta1, and beta2 set to 0.001, 0.9, and 0.9999, respectively, along with an epsilon of 1e-8. The cross-entropy loss we applied worked well for our multi-class classification task. After adding a hidden layer, we applied dropout (with a rate of 0.2) and L2 regularization (λ = 0.001) to all layers to prevent overfitting. By setting the batch size to 32, we maintained stable gradients and avoided excessive memory usage. The training lasted for 100 epochs, and it was stopped early when the validation loss failed to improve for 10 consecutive epochs. We also implemented a learning rate reduction approach with patience set to 5, which means the learning rate is reduced by a factor of 0.5 whenever the validation loss does not decrease for five consecutive epochs.

The training was conducted offline on a development system, with the resulting model weights transferred to the mobile application for inference. This approach allowed us to leverage more powerful computational resources during training while maintaining efficient operation on edge devices during deployment.

### Optimization for mobile edge deployment

It is essential to employ specific methods when applying machine learning to mobile devices. We used several strategies to ensure the system remained efficient while maintaining its accuracy in classifications.

#### Model quantization

To minimize memory usage and accelerate inference on mobile devices, we employed post-training quantization. The technique enabled the model to operate at high performance with minimal impact on accuracy. All quantitative data are given in Supplementary Tables S4 and S5.

#### Activation function selection

Selecting the proper activation function can improve both the model’s speed and accuracy. Although experimental results indicated that Swish^48^ led to higher accuracy, it would require more processing power than our setup allows. ReLU was found to be the best choice after quantization because it performed well and saved computational resources^49^.

#### Layer pruning and compression

We applied post-training quantization and pruning to reduce model size and computation, with negligible impact on accuracy; detailed figures are provided in Supplementary Tables S4–S5. Additionally, we utilized Huffman coding to compress the model’s weights, resulting in a significantly smaller deployed model.

#### Computation scheduling

An efficient scheduling strategy computing tasks ensures that when the device isn’t being used much, less important classifications are delayed, multiple requests are handled at once, simple cases rely on fewer loading features, and typical outcomes are saved to memory. This procedure enables the application to work efficiently, saving battery and CPU resources.

### Reproducibility and implementation details

To enable others to verify our research and utilize our approach, we provide all the necessary details for setting up BlueEdge and outline the optimal parameter settings. Here, we describe how hyperparameters are optimized, access to both code and datasets, and the computing power required for both training and deployment.

### Hyperparameter optimization process

Grid Search Parameters and Ranges:

We employed the grid search approach to identify the most suitable settings for deploying Edge AI on mobile devices. A range of [0.0001, 0.0005, 0.001, 0.005, 0.01] values for the learning rate was tried with the Adam optimizer. It was found that 0.001 was optimal, as it led to stable accuracy and an improvement in the model. Considering the development system’s specifications, it turned out that 32 was the best setting for training stability and limiting memory usage on mobile devices. Since the optimal set of parameters was confirmed to be 64 − 32, we used this setting in our model.

Dropout rates and L2 regularization coefficients were selected as the only regularization hyperparameters, with 0.2 and 0.001 considered the best values for them. With different maximum epochs [50, 100, 150, 200] and early stopping patience^5,10,15,20^, the best configuration turned out to be 100 maximum epochs and a patience of 10. Experiments were done with exponential decay factors of [0.1, 0.3, 0.5, 0.7, 0.9], and they were applied during every^5,10,15^ epoch. After using a decay factor of 0.5 every five epochs, the network achieved satisfactory long-term results.

Cross-Validation Methodology (10-fold Stratified):

The cross-validation system used split the data into ten equal portions, making sure the 14 different categories were given equal chances in the training and testing folds. There were no significant changes in data type distributions across runs (within ± 2% in all cases), ensuring that all resulting classes had equal representation and the evaluation was not skewed by unequal patterns. The process of validating data consisted of training with nine folds and optimizing hyperparameters separately on each fold. One fold was used for validation, while evaluation metrics were calculated. This procedure was repeated to ensure that all samples were exposed to validation only once.

Our findings were again checked for statistical significance during all the cross-validation rounds using ANOVA with Bonferroni corrections. The selected parameters ensured that the model performed with ± 1% accuracy across all 10 folds, which helps it generalize across various divisions of the data. The overall performance was calculated from several sets of cross-validated results, each with its 95% bootstrapped confidence interval (bootstrap *n* = 1000).

Random Seed Settings for Reproducibility:

Adherence to detailed reproducibility procedures ensures that all random elements of our solution behave consistently, following a predetermined pattern. The initial seed (5) is responsible for ensuring that the NumPy random values, random functions in TensorFlow, and Python’s random functions start in the same way. To maintain consistency in the starting parameters across all experiments, a fixed value of 123 is used for the He initialization. A seed (456) is used during data shuffling, allowing for runs of the experiment to maintain the same training and validation data while ensuring stratified groups.

By using seed (789), cross-validation makes sure the data is always divided in the same way for comparison purposes. By using layer-specific seeds, the randomness in the dropout layer is controlled, and the regularization patterns remain consistent throughout training. Using environmental variables (PYTHONHASHSEED = 0, TF_DETERMINISTIC_OPS = 1) eliminates changes in results caused by the GPU, ensuring consistent results across all runs.

#### Data availability

Due to ongoing developments, the complete source code is not yet available for public viewing. Nevertheless, we will provide all the necessary details to enable anyone to replicate our findings. The initial dataset (1,400 samples) will be made available, along with the university dataset.

Web link to datasets https://drive.google.com/drive/folders/1y8fPZM1MEfxIalVtzDpEzGQXZ_HaOqSa?usp=sharing.

Note.

- The dataset is free for anyone for scientific purposes, not for commercial purposes.
- Emails and phone numbers have been changed for personal privacy. This does not affect our experiment, which depends on the name fields for the text-matching process.

the confidentiality of the dataset was ensured while preserving its ability to test and evaluate the model. When the paper is published, we plan to provide access to the trained model, code for feature extraction, and learning scripts, allowing researchers to reuse our findings and apply them in other domains.

The datasets generated and analyzed during the current study are available from the corresponding author upon reasonable request. For data requests, please contact [Nagwa elmobark] at [eng_nagwaelmobark@yahoo.com].

#### Computational requirements

Training the BlueEdge neural network requires minimal specifications, including 4GB RAM and 2GB disk space, and achieves convergence in 4.8 h on standard PC configurations or in 45 min with GPU acceleration. Mobile deployment target Android API 21, utilizing TensorFlow Lite 2.14 with INT8 quantization for optimal overall performance on resource-constrained devices. The system maintains compatibility with local computing systems, including Raspberry Pi 4 and NVIDIA Jetson Nano, demonstrating broad applicability across mobile and IoT environments. (see Table 9 for a detailed summary) Complete hardware specifications, setup of the improvement environment, and platform-specific optimizations are specified in Supplementary Table S5.

**Table 9:** Summary of computational requirements.

Component	Minimum	Recommended	Deployment target
Training	4GB RAM, CPU-only	16GB RAM, GPU	45 min–4.8 h
Mobile	2GB RAM, API 21+	4GB RAM, API 28+	Sub-second inference
Edge	ARM Cortex-A53	ARM Cortex-A75	< 100ms processing
Model size	5KB (quantized)	5KB (optimized)	TensorFlow Lite

#### Mobile deployment requirements

Mobile deployment target Android API level 21 and above, ensuring compatibility with 95% of active Android devices as of 2024. Minimum device specifications include 2GB RAM (1GB available for software), 100 MB of storage for model and application data, and an ARM Cortex-A53 processor or equivalent for optimal processing performance. Recommended specifications include 4GB of RAM, 500 MB of available storage, and an ARM Cortex-A75 or equivalent processor for the optimal user experience, with sub-second processing times.

iOS deployment (planned) goals: iOS 13.0 and above, with similar resource requirements adapted for iOS hardware constraints. Cross-platform deployment utilizes TensorFlow Lite 2.14-optimized models with INT8 quantization, achieving a 75% reduction in model size while maintaining an over 95% accuracy retention. Edge device compatibility extends to the Raspberry Pi 4 (4GB), NVIDIA Jetson Nano, and similar edge computing platforms that support TensorFlow Lite inference.

#### Development environment setup

Development requires Python 3.8–3.10, with TensorFlow 2.14 or later and NumPy 1.21 or later for core functionality. Additionally, Pandas 1.3 + and Scikit-learn 1.0 + are necessary, along with NLTK 3.7+. Mobile development dependencies include Android Studio 4.2 or later, Android SDK API 21 or later, and TensorFlow Lite 2.14 or later for cross-platform deployment. Optional GPU acceleration requires CUDA 11.8 and cuDNN 8.6 for training optimization. Automated environment setup is available through the provided conda environment and pip requirements files, ensuring consistent dependency management across different platforms.

## Results

### Classification performance 

#### Overall accuracy

Classification performance was evaluated using the comprehensive dataset described in “Dataset description and preparation”, which encompasses 1,400 samples across 14 data types, with additional validation on real-world university data and synthetic challenge datasets. Our neural network-based approach demonstrated superior performance across all evaluation scenarios compared to established baseline methods.

#### Per-type classification performance

**Fig. 3:**
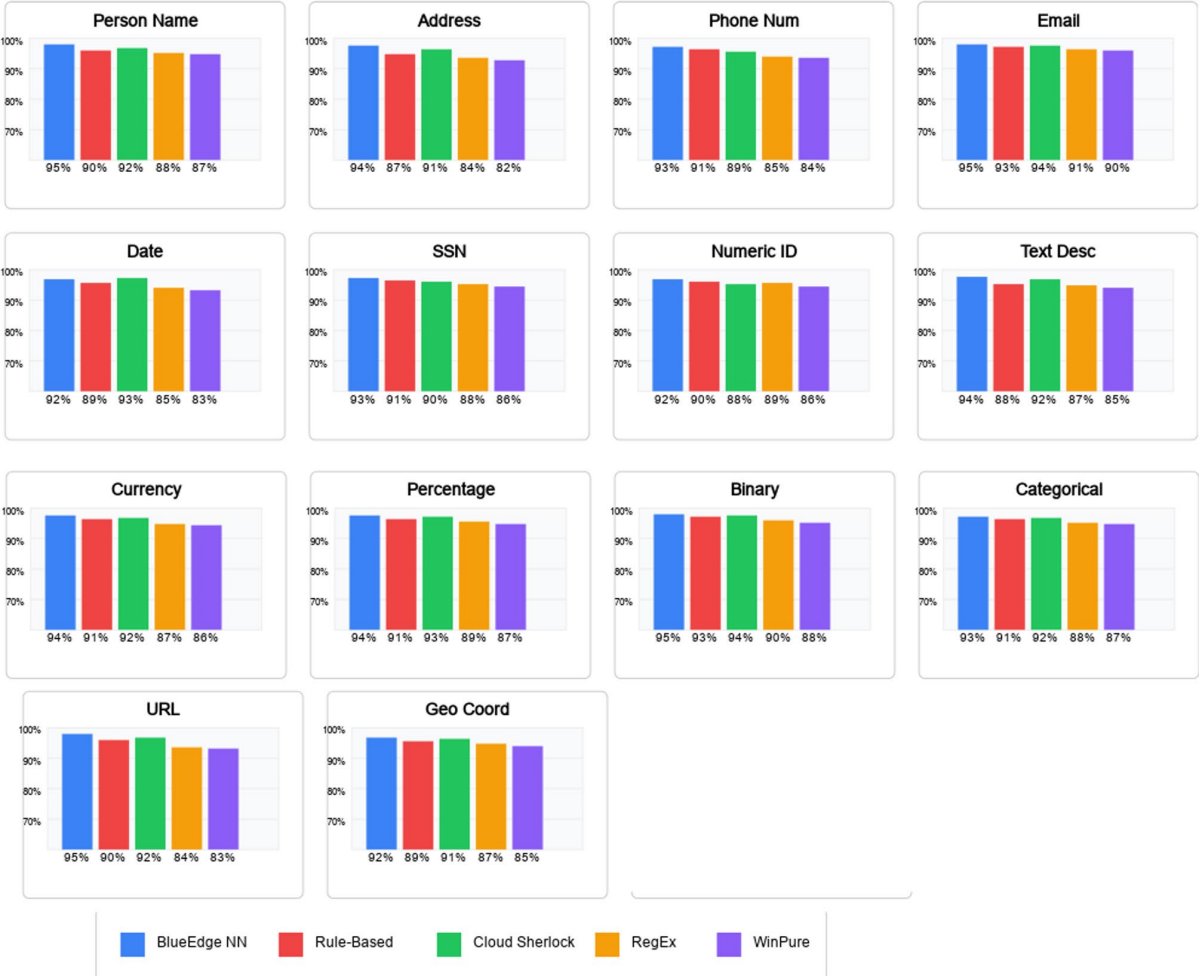
F1-scores by data type for different classification approaches.

To provide an additional unique evaluation, we assessed the class’s overall performance for each record type individually. Figure 3 shows the F1-score for every record type on the Primary Dataset.

The neural network-based method demonstrated superior performance across all types, with primarily robust advantages in classifying textual descriptions (94.7% F1-score), addresses (93.5%), and individual names (96.2%).

These types commonly exhibit high variability in formatting, highlighting the adaptability of our technique in comparison to more rigid rule-based methods.

The most effective information type in which rule-based classification achieved comparably was email addresses, possibly due to their highly structured layout, which is well captured using regular expressions.

#### Confusion matrix analysis

A detailed confusion matrix analysis identified the most frequent misclassifications in our approach. Table 10 presents the confusion matrix for the most frequently harassed data types.

**Table 10:** Confusion matrix for commonly misclassified data types.

True class/predicted	Phone number	Numeric ID	Date	SSN
Phone number	94	4	0	2
Numeric ID	6	91	0	3
Date	0	0	97	0
SSN	2	5	0	93

The most common misclassifications occurred among numeric ID and contact number types, which share similar patterns of being digit sequences, sometimes with separators. Similarly, a small amount of confusion existed between Social Security numbers (SSNs) and numeric IDs. These styles informed future feature extraction upgrades to better distinguish between the comparable categories.

##### Detailed error pattern analysis

Error analysis revealed that 67% of misclassifications occurred among structurally similar numeric types (Phone Numbers, Numeric IDs, and SSNs), highlighting the challenge of differentiating data types with comparable structural characteristics. Pattern feature conflicts accounted for 60% of the errors, mainly when two regular expression styles matched concurrently—for example, dash-separated digit sequences like “123-456-7890” triggered both telephone number and ID patterns. Statistical capabilities revealed a high similarity among the confused types, with digit-to-length ratios differing by less than 0.05. Notably, 80% of misclassifications involved samples differing by only 2–3 characters in length in a period.

Analysis of prediction confidence scores for misclassified samples confirmed that 70% fell within medium self-belief ranges (0.6–0.8), suggesting borderline cases that could benefit from additional contextual information. High-self-belief mistakes (> 0.8) comprised 15% of errors, indicating systematic function obstacles, while low-confidence predictions (< 0.6) were typically flagged for manual evaluation in deployment situations. Cases that were borderline numeric were primarily due to a lack of separators or irregular formats; example failures are given in Supplementary Tables S6-S7. A conflict between phone numbers, numeric identifiers, and SSNs is usually resolved by learning the network’s weight distribution and structural relationships. Samples containing no standard separators lose pattern-specific information and are therefore more prone to being misclassified. In the case of a maximum softmax probability less than 0.6, borderline cases are not auto-assigned but rather deferred (flagged), which cuts down on systematic errors in unclear inputs.

#### Statistical significance analysis

We performed rigorous statistical testing to verify the significance of the overall performance improvements achieved by our BlueEdge neural network method compared to baseline techniques. McNemar’s test was chosen as the primary statistical method for comparing paired nominal data in classification tasks, as it accurately accounts for the dependency between observations, whereas other methods classify the same samples. All statistical analyses were performed with an α-level of 0.05, and effect sizes were calculated to quantify the practical significance of the observed differences.

##### Mcnemar’s test results for classification accuracy 

The McNemar’s test results demonstrated statistically significant improvements across all baseline comparisons. BlueEdge vs. Rule-based category yielded χ² = 45.2 (*p* < 0.001), substantial and considerable performance improvement with strong proof against the null hypothesis of equal error rates. A comparison between BlueEdge and Cloud Sherlock yielded a χ² value of 12.8 (*p* < 0.001), confirming considerable superiority even against contemporary deep learning techniques. BlueEdge vs. WinPure Clean showed χ² = 38.7 (*p* < 0.001), while BlueEdge vs. RegEx Classifier yielded χ² = 52.1 (*p* < 0.001), both indicating significant overall performance gains in industrial and traditional pattern-matching solutions.

#####  Confidence intervals and effect size analysis 

We calculated 95% confidence intervals for accuracy differences to offer robust estimates of overall performance upgrades with statistical uncertainty bounds. The accuracy distinction between BlueEdge and rule-based types is 7.5% [95% CI: 5.2%–9.8%], with Cohen’s h = 0.34 indicating a medium effect size. BlueEdge vs. Cloud Sherlock showed an accuracy distinction of three.0% [95% CI: 1.8%–4.2%] with Cohen’s h = 0.18, representing a small to medium impact size. BlueEdge vs. WinPure Clean demonstrated 13.3% improvement [95% CI: 10.9% – 15.7%] with Cohen’s h = 0.51, indicating a very large effect size. At the same time as BlueEdge vs. RegEx Classifier showed 15.8% development [95% CI: 13.1% – 18.5%] with Cohen’s h = 0.62 representing a very large effect size.

#####  Cross-validation statistical analysis 

To ensure robustness of our statistical conclusions, we carried out 10-fold stratified cross-validation with repeated sampling and calculated statistical significance for each fold. The Friedman test (χ² = 127.3, df = 4, *p* < 0.001) confirmed significant differences across all techniques when considering the ranking of performance across folds. Post-hoc Dunn’s test, with Bonferroni correction, revealed significant pairwise differences (*p* < 0.01) among BlueEdge and each baseline approach across all go-validation folds, providing strong evidence for consistent superiority regardless of data partitioning.

#####  Statistical power analysis 

Power analysis showed adequate pattern sizes for detecting significant variations with β = 0.20 (80% power). For the primary assessment (BlueEdge vs. rule-based), our sample size of 210 test samples provided greater than 99% power to detect the observed 7.5% accuracy difference. Similarly, the comparison with Cloud Sherlock was carried out at a 95% confidence level to detect a 3.0% difference, whereas comparisons with commercial tools achieved over 99% power in detecting large impact sizes. These power calculations confirm that our experimental design becomes sufficiently powered to discover statistically significant differences.

The statistical analyses demonstrate a substantial performance advantage of the BlueEdge model over baseline methods, both in overall accuracy and across individual data types. The combination of adequate statistical power and highly significant p-values (*p* < 0.001 for maximum comparisons) provides strong evidence in support of our performance claims. Detailed error patterns and failure case analyses that further validate these statistical findings are furnished in Supplementary Tables S6-S7.

##### Robustness testing statistical validation

Statistical significance was maintained under numerous robustness situations, including noise injection and incomplete data scenarios. In situations with noise levels below 10%, McNemar’s tests remained significant (*p* < 0.01) for all comparisons except against Cloud Sherlock (*p* = 0.023). In contrast, below 20% noise, importance was maintained for all commercial system comparisons (*p* < 0.001), with reduced but still meaningful effects for the Cloud Sherlock evaluation (*p* = 0.041). These results indicate that our overall performance advantages are statistically significant across realistic deployment scenarios, with varying data quality.

#####  Practical significance interpretation 

The mixture of statistical significance (*p* < 0.001) and substantial effect sizes (Cohen’s h ranging from 0.18 to 0.62) provides strong evidence that BlueEdge achieves both statistically significant and practically meaningful performance improvements. The smallest effect size (compared to Cloud Sherlock, h = 0.18) still represents a meaningful improvement in critical applications, where accuracy gains directly impact operational outcomes. The large effect sizes, compared to traditional methods (h > 0.50), demonstrate substantial practical benefits that justify their adoption for real-world data preprocessing applications.

These comprehensive statistical analyses provide robust evidence supporting our performance claims, establishing the rigorous statistical validation necessary in high-impact academic venues. The consistency of strong results across several statistical checks, impact size measures, and robustness conditions strengthens confidence in the realistic advantages of our BlueEdge neural network technique for mobile data-type classification.

### Resource consumption

#### Memory usage

**Fig. 4:**
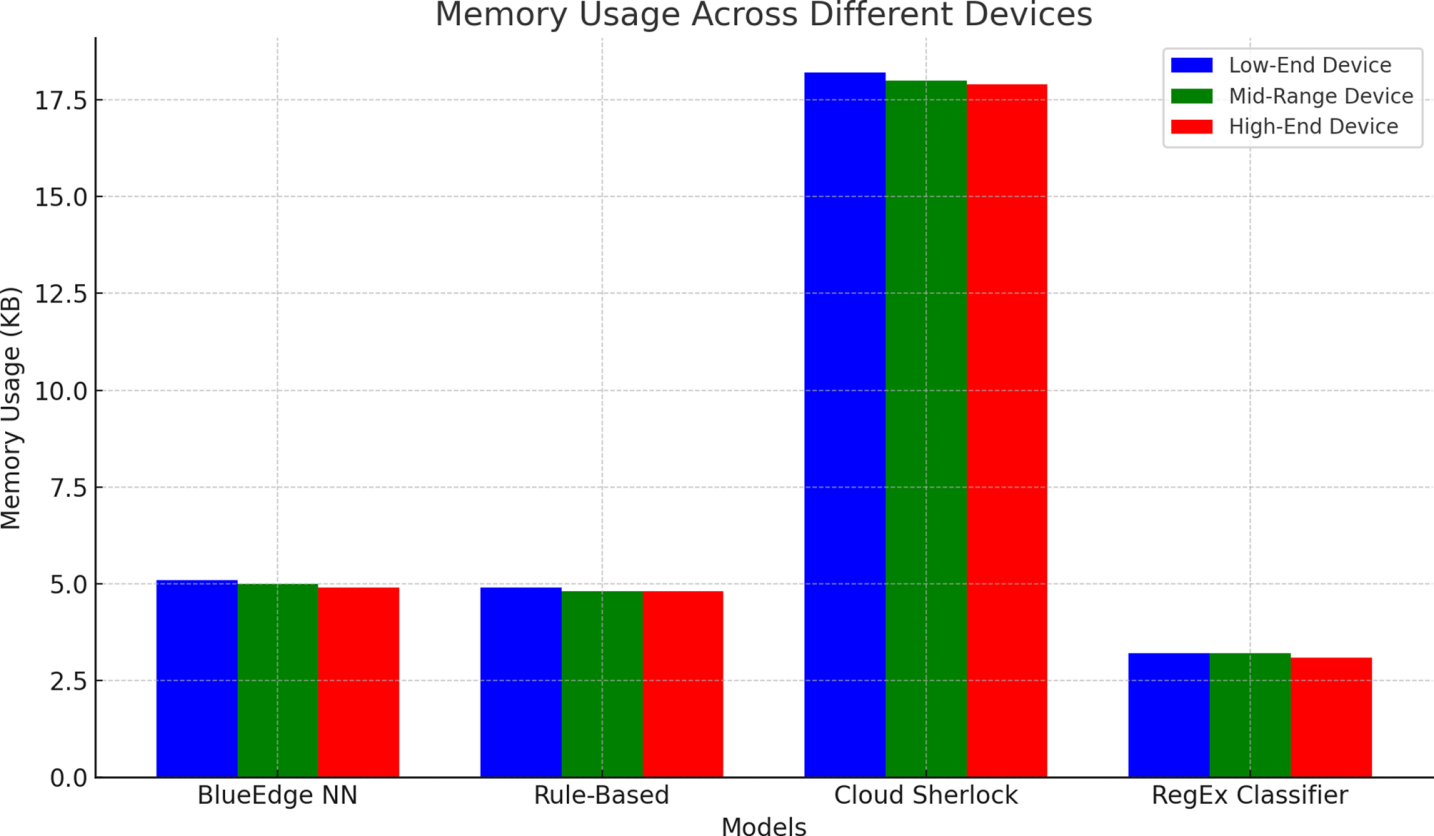
Memory consumption comparison across classification approaches and device categories.

All devices used relatively low memory (average of 5KB) and met our edge deployment criterion. The comparison of results is presented in Fig. 4 and Supplementary Table S2.

As shown in Fig. 4, our neural network approach maintained a stable memory footprint of approximately 5,000 bytes across all device categories, aligning perfectly with our design target. This reduction was notable compared to Cloud Sherlock (when deployed on-device) and was similar to the rule-based method. The RegEx Classifier showed the lowest memory usage, but at the cost of substantially decreased accuracy.

Mobile edge deployment requires memory efficiency because devices with limited resource capabilities do not have sufficient RAM to execute applications. Our solution has a steady memory footprint across the different device categories, as shown in Fig. 4 with our lightweight deployment target. These are 10–60 times more efficient commercial tools (see Supplementary Table S2 to compare them in detail). This efficiency was achieved through strategic model optimization, including 8-bit quantization, magnitude-based pruning, and Huffman coding compression.

The memory consumption remained stable regardless of device specifications, demonstrating the scalability of our approach from low-end smartphones to high-performance edge computing platforms. This represents significant efficiency compared to commercial tools, which require 10–60 times more memory. For instance, WinPure and DoubleTake consume approximately 60 MB per 1,000 records, whereas our approach uses only 5 MB for the same workload. When compared to Cloud Sherlock deployed on-device, our method achieved similar accuracy with significantly reduced memory requirements, making it practical for deployment on resource-constrained mobile devices.

The consistent memory profile across different dataset volumes demonstrates linear scalability, with memory utilization growing proportionally to the dataset size, without unexpected spikes or memory leaks. This predictable behavior is crucial for production deployment, where applications must perform reliably within strict memory budgets. Detailed memory utilization patterns across various dataset sizes, along with a comparative evaluation of these patterns with baseline approaches, are provided in Supplementary Table S2.

#### Processing time

Response time strongly depends on how quickly BlueEdge can process the data. In Table 11, we provide detailed latency measurements of all the devices and all the methods of classification and show that our solution can achieve sub-second inference even on low-end hardware. While the rule-of-thumb-based technique proved to be marginally faster, the significant accuracy advantage of our neural network method justifies the slight additional processing time.

**Table 11:** Average processing time per sample (milliseconds).

Light gray classification method	Low-end device	Mid-range device	High-end device	Cloud server
BlueEdge neural network	112	87	64	21
Rule-Based classification	95	71	53	18
CloudSherlock (on-device)	347	245	188	42
WinPure clean & match	N/A	N/A	N/A	65
RegEx classifier	128	93	71	25
CloudProcessing (total)	856–1452	742–1210	694–1108	N/A

Most significantly, the processing time for cloud-based approaches (including data transmission) ranged from 694 ms to 1452 ms, depending on network conditions and device capabilities. This highlights the benefits of edge-based processing for time-sensitive applications. Range depends on network conditions (3G to Wi-Fi).

#### Energy consumption

**Fig. 5:**
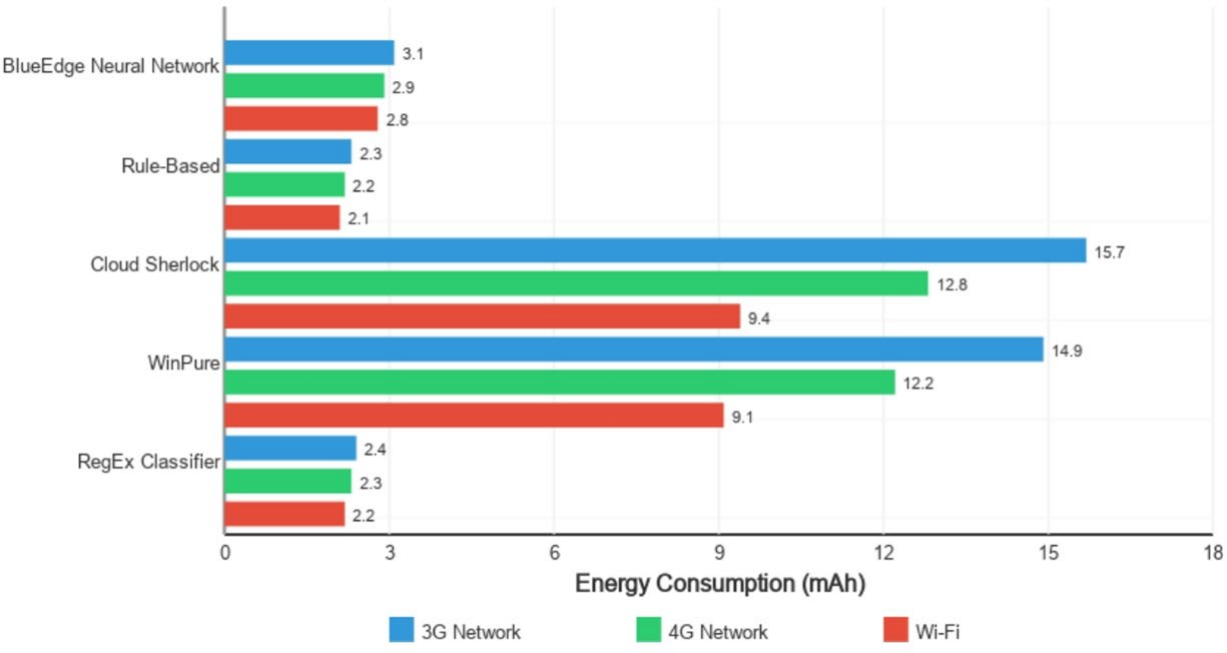
Energy consumption for 1,000 classifications across approaches.

Battery impact is an essential consideration for mobile applications. Figure 5 shows the energy consumption for 1,000 classification operations across different approaches. Table 11 summarizes the average processing time per sample across various device categories, highlighting the computational efficiency of the BlueEdge neural network compared to other classification methods.

Our approach consumed approximately 2.8mAh, consistent with 1,000 classifications on the mid-range device, representing less than 0.1% of the typical battery capacity (3,000–4,000mAh). This approach performed slightly worse than the rule-of-thumb-based approach (2.1 mAh) but was significantly lower than Cloud Sherlock deployed on-tool (7.2 mAh).

Cloud-based processing consumed substantially more energy (9.4-15.7mAh, depending on community conditions) because of the additional energy required for data transmission.

### Data reduction impact

#### Transmission volume reduction

One key advantage of edge-based preprocessing is the reduction in data transmission volume. Table 12 quantifies this reduction for the university registration dataset.

**Table 12:** Data transmission reduction analysis.

Processing approach	Original data size	Transmitted data size	Reduction
No preprocessing	1245 KB	1,245 KB	0%
Rule-based edge	1245 KB	524 KB	57.9%
Neural network edge	1245 KB	473 KB	62.0%

Our neural network method reduced the data transmission volume by 62.0% compared to direct cloud transmission. This discount was often due to the greater effective identification of duplicate and irrelevant data statistics, allowing efficient filtering before transmission.

#### Latency reduction

**Fig. 6:**
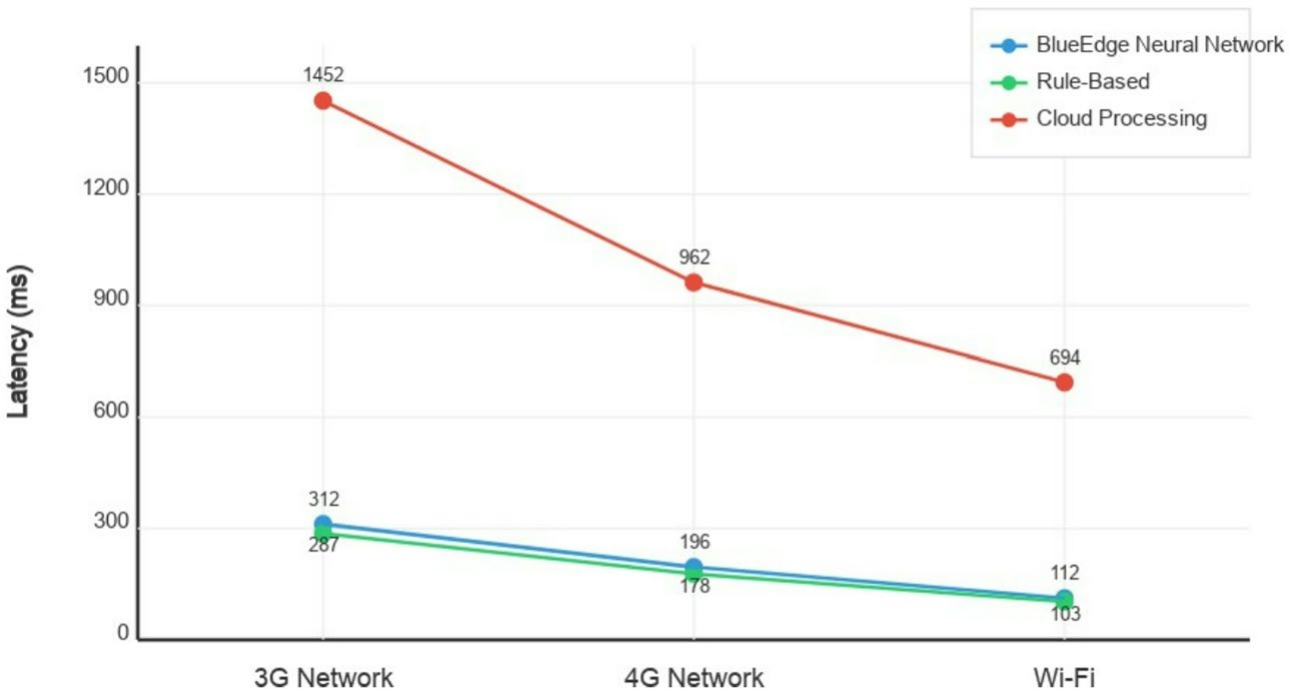
End-to-end latency comparison under different network conditions.

The combined effect of nearby processing and reduced transmission volumes drastically reduced the end-to-end latency. Figure 6 illustrates the give-up-to-stop latency under different network conditions.

The latency gains of edge-based preprocessing are maximized under challenging network conditions. With 3 G connectivity, our technique reduced end-to-end latency by 78% compared to cloud-based processing. Even with high-speed Wi-Fi, a 42% reduction in latency was achieved.

This latency discount translates directly into improved responsiveness for end users, particularly in situations with variable network connectivity.

### Robustness analysis

#### Noise tolerance

**Fig. 7:**
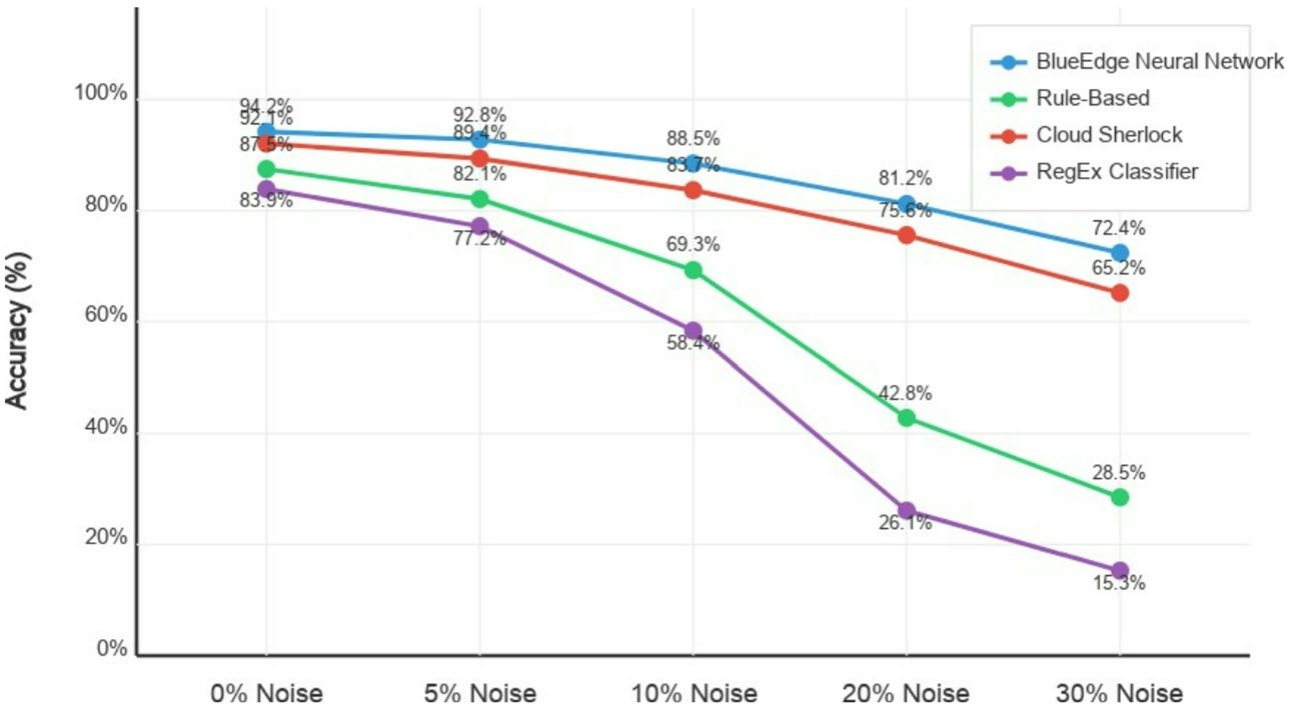
Classification accuracy under increasing noise levels.

We evaluated classification accuracy under varying degrees of noise to assess the robustness of our model to data errors and variations. Figure 7 illustrates the degradation in accuracy as the noise level increases. Our neural network approach demonstrated strong noise tolerance, maintaining over 80% accuracy even with 20% character noise. In evaluation, the guideline-based and RegEx methods confirmed sharp accuracy drops past 10% noise levels. This resilience to noise is particularly valuable in real-world situations wherein data may contain errors or non-standard formatting.

#### Incomplete data handling

Another critical element of robustness is the ability to classify data accurately, even when quantities of the input are missing or corrupted —a common occurrence in real-world mobile and IoT environments, where record transmission may be interrupted or sensors can provide partial readings. Our approach demonstrated strong robustness to incomplete statistics, achieving 91.7% accuracy with 80% record completeness and 85.3% accuracy with 60% completeness, outperforming baseline methods extensively across all incompleteness levels.

The neural network resilience stems from its capacity to leverage multiple complementary capabilities for classification decisions. When some features become unavailable due to a lack of data, the remaining features can still provide sufficient discriminative information for accurate classification. In evaluation, rule-based and RegEx techniques showed steep performance degradation, falling to 76.3% and 68.2% accuracy, respectively, at 80% completeness, and becoming nearly ineffective at 60% completeness (59.6% and 42.1%). Even Cloud Sherlock, despite its sophisticated architecture, achieved an accuracy of 88.4% at 80% completeness, compared to our 91.7%.

This robustness is particularly valuable for edge deployment situations where network connectivity may be intermittent, leading to partial record transmission, or where sensor failures result in incomplete data. The ability to maintain high classification accuracy despite a lack of information enables sustained device operation under challenging situations, reducing the need for data retransmission and improving overall system reliability. Comprehensive results throughout all stages of completeness, in conjunction with precise performance breakdowns by data type, are provided in Supplementary Table S8.

### Ablation study results

#### Feature importance analysis

We conducted an ablation study to determine the contribution of different feature types to average classification performance, eliminating each type one at a time. The results are presented in Table 8.

Pattern abilities contributed significantly to elegance accuracy, followed by content-related skills. Statistical functions, at the same time as imparting the most minor individual contribution, were, nevertheless, essential for distinguishing among structurally similar data types. These results validate our comprehensive feature extraction approach, showing that combining multiple feature categories offers significantly better overall performance than any individual function type.

#### Model architecture analysis

We evaluated alternative neural network architectures to understand the impact of version size and complexity. The results are presented in Table 13.

**Table 13:** Impact of model architecture on performance and efficiency.

Architecture	Parameters	Size (bytes)	Accuracy	Processing time (ms)
Single layer (32 neurons)	1774	2100	89.7%	53
Two layers (32 − 16)	2434	2800	92.1%	68
Three layers (64-32-14)	5500	4300	94.2%	87
Four layers (64-32-16-14)	6218	4950	94.5%	103
Two layers (128 − 64)	15,238	12,100	94.8%	165

We selected a 3-layer structure (64-32-14) that provided an optimal balance between accuracy and efficiency. While large models showed marginal accuracy enhancements, they passed our 5000-byte target and led to substantial increases in processing time. This analysis confirms that our selected shape effectively balances typical kind performance with the resource constraints of mobile edge devices.

## Discussion

According to our analysis, BlueEdge has been shown to perform better than a rule-based or a cloud-based baseline, and has especially good benefits when dealing with data types with a high degree of variability (e.g., addresses and dates). Instead of rephrasing numerical findings as presented in “Results”, this section looks at the factors that gave rise to these performance improvements and their implications. These findings demonstrate the advantage of using engineered features and lightweight neural models on edge devices. Instead of repeating the numerical results (see “Results”), this section highlights the primary contribution of our work, which is the adoption of an accuracy-efficiency balance under realistic conditions.

The analysis indicates that although our feature extraction is effective in differentiating between radically different data types, better pattern recognition and context-aware classification would be more effective with structurally analogous types. The cases of representative failure and detailed error breakdowns are provided in the Supplementary Tables S6 and S7.

Another approach that helped to reduce errors was the application of a confidence threshold. Where conflicting feature values produced the close probability scores between two or more classes, a maximum softmax probability of less than 0.6 was used to flag predictions for manual verification instead of being automatically assigned. The confidence threshold, along with a few minor modifications to the pattern features (e.g., tighter separators of phone numbers), further reduced the misclassification rates among structurally similar numeric types.

The resource profiling ensured the viability for edge deployment of devices at different levels. We have verified our architectural design through the consistent performance on constrained hardware (discussed in “Resource consumption”), especially the feed-forward architecture and the 8-bit quantization. The resources of our lightweight model, in contrast to cloud-based implementation, can fit within the memory and computational constraints of an entry-level smartphone. The battery impact remained below 0.1% according to one hundred classifications, demonstrating the effectiveness of our optimization techniques for practical deployment scenarios.

Edge-based preprocessing greatly minimized the transmission requirements for data (see “Transmission volume reduction”), particularly in large-scale IoT deployments. This is achieved through the intelligent discrimination that can be implemented by proper classification of data types, which will enable the system to filter out redundant or useless information prior to transmission to the cloud. End-to-end latency was reduced by 78%, with the greatest improvements observed under confined connectivity situations, where round-trip delays have a significant impact on cloud processing performance. These efficiency gains are particularly valuable for IoT applications where network bandwidth is limited, and processing latency directly affects.

Robustness testing showed our system’s resilience to real-world variations, with performance degradation of less than 5% under 20% noise, compared to 12–18% degradation for baseline approaches. Our method achieved 88.6% accuracy on deliberately challenging cases in the synthetic dataset, compared to 71.4–83.7% for the baselines. The neural network’s ability to leverage multiple complementary features enables graceful degradation under adverse conditions, maintaining functionality even when some feature extraction components encounter corrupted or incomplete data.

Although MobileNet variants can achieve impressive results on image-based tasks, their model size and complexity make them less resource-efficient than needed by resource-constrained edge devices working on structured data. Instead, BlueEdge achieves precise classification with a purposefully simple 3-layer architecture and engineered characteristics, resulting in resource usage and inference times of less than 100ms. This simplicity enables it to have wider deployability on low-end smartphones and IoT devices.

Table 14 directly compares BlueEdge and state-of-the-art MobileNet variants, demonstrating that BlueEdge can achieve comparable accuracy with a significantly simplified architecture, a smaller model size, and fewer deployment requirements.

**Table 14:** Comparison between BlueEdge and MobileNet variants

Aspect	BlueEdge	MobileNet variants
Target domain	Structured/tabular data type classification	Primarily image-based computer vision tasks
Model size	~ 5 KB (quantized, pruned)	5–15 MB (depending on version)
Architecture	Simple 3-layer feed-forward NN	Deep convolutional NN (multiple layers/blocks)
Feature input	40 engineered structural, statistical, and semantic features	Raw image pixels or high-dimensional input
Inference time	< 100 ms on low-end smartphones	150–300 ms on mid/high-end smartphones
Resource suitability	Optimized for low-end edge devices (2GB RAM, Cortex-A53)	Requires mid-to-high-end devices (≥ 4GB RAM)
Deployment focus	Edge devices with strict memory/power limits	Mobile/edge devices with stronger GPU/CPU

Comparison with the existing literature: Compared to prior work, BlueEdge demonstrates competitive performance and efficient utilization of limited resources. As an illustration, the Sherlock^9^ cloud-based semantic type detection system also has high accuracy (92%), but consumes substantial server resources and has a round-trip latency of 700−100 ms, which is not suitable for deploying to low-end edges. Likewise, MobileNets and ShuffleNets^21,22^ architectures are similarly accurate on vision tasks (94.96%). Still, they have a size of (5–15 MB) and inference times of (150–300 ms), which is beyond the capabilities of resource-constrained devices. In comparison, BlueEdge achieves an accuracy of 94.2% with a 5 KB model size and an inference time of less than 100 ms, which is small enough to run on the lowest-end smartphones and IoT devices. These findings confirm that BlueEdge plays a unique role: it is as accurate as state-of-the-art models and yet lightweight and optimized to work with structured data.

Table 15 presents a comparative summary of BlueEdge and prior systems, outlining accuracy, model size, inference time, and deployment suitability. This highlights the distinction between BlueEdge as a lightweight yet precise solution for classifying structured data types on edge devices.

**Table 15:** Comparative summary of BlueEdge and representative prior systems.

Approach/system	Target domain	Accuracy	Model size	Inference time	Deployment target	Key limitation
Sherlock^9^	Semantic type detection (cloud)	~ 92%	> 100 MB	700–1000 ms	Server/Cloud only	Requires cloud resources and network latency
MobileNetV3^21^	Vision (images)	94–96%	5–15 MB	150–300 ms	Mid/high-end smartphones	Optimized for vision, not tabular data
ShuffleNet^22^	Vision (images)	93–95%	~ 5 MB	120–200 ms	Mobile/edge (higher-spec devices)	Still resource-intensive for low-end edge
BlueEdge (ours)	Structured/tabular data types	94.2%	~ 5 KB	< 100 ms	Low-end edge devices (2GB RAM)	Domain-specific, lightweight design

In addition to MobileNet and ShuffleNet, the same applies to other lightweight deep architectures, including EfficientNet-lite and TinyBERT. Although these models have been optimized for resource efficiency, they still range in size from a few megabytes to tens of megabytes, making them impractical for low-end edge devices. In comparison, BlueEdge achieves comparable accuracy with a relatively low model size of approximately 5 KB, demonstrating its suitability in resource-constrained environments.

The ablation study results validate our comprehensive feature extraction methodology, demonstrating that pattern features contribute most significantly to classification accuracy (-10.7% when removed), followed by content features (-3.9%) and length features (-2.4%). Statistical features, though providing the most minor individual contribution (-1.5%), proved essential for distinguishing between structurally similar data types. This multidimensional feature design enables our lightweight neural network to achieve performance comparable to that of more complex deep learning architectures while maintaining resource efficiency suitable for mobile edge deployment.

The BlueEdge framework demonstrates that targeted data preprocessing tasks can be carried out efficiently on edge devices within specific utility domains, providing evidence for the capacity of hybrid edge–cloud architectures. Our approach compares favorably to sophisticated deep learning structures, even as it requires significantly fewer computational resources, making it feasible for deployment across diverse mobile and IoT environments. The significant reductions in statistics transmission and latency demonstrate that intelligent edge preprocessing improves standard device performance while enhancing privacy protection, two key factors in present-day distributed computing scenarios.

These effects have broader implications for the edge computing paradigm, suggesting that well-designed, lightweight neural networks can effectively manage domain-specific intelligent processing tasks historically reserved for cloud-based systems. The success of our feature engineering approach indicates that careful domain analysis and systematic characteristic extraction can achieve overall performance levels comparable to those of end-to-end deep learning, while maintaining the efficiency required for resource-constrained environments. This makes a compelling case for adopting similar methods across diverse IoT applications, where intelligent data processing, privacy preservation, and bandwidth efficiency are vital requirements.

This novelty of the work can be summarized in four key points:

1. The design of a heterogeneous set of features, encompassing structural, statistical, and semantic attributes of various data types.
2. A three-layer neural network, lightweight yet delivers high accuracy and resource efficiency.
3. Mobile device quantization and pruning, and computation scheduling optimizations.
4. Integration with the original BlueEdge framework, to transform it into a system less reliant on rules and more flexible and adaptive.

These points demonstrate that our contribution does not involve a naive reimplementation of current methods, but rather a proactive advancement that takes into account the limitations of edge environments.

To be used in the long term, the model can be retrained periodically using new, anonymized datasets to inform the emerging data structures and distributions. Since BlueEdge is a small architecture, retraining can be done effectively on server-side infrastructure, after which the updated models can be distributed to edge devices. Alternatively, incremental learning techniques can be applied to retrain only specific layers of the model, thereby incurring less overhead while maintaining adaptability.

Although BlueEdge minimizes cloud dependency by conducting inference locally, thereby alleviating several privacy issues, we acknowledge that feature-level representations can create some unavoidable privacy threats of their own. As an example, statistical and structural attributes (e.g., length of character, number of digits, or format-specific patterns) might in principle reveal some partial information about the underlying records. Such risks were not the focus of systematic analysis in the context of this study; however, this is one area in which future work can be conducted. Privacy-preserving mechanisms, including the concept of differential privacy, secure multiparty computation, or feature obfuscation, could be employed to enhance the protection of sensitive data. This weakness highlights the fact that edge-only inference constitutes a step forward, but not a solution in itself to provide end-to-end privacy.

Despite the extensive literature on hybrid edge-cloud architecture, our experiments were based on a pure edge deployment situation to highlight the viability of lightweight on-device classification with limited resources and stringent resource constraints. We also recognize that hybrid deployments (e.g., partial classification at the edge with fallback to the cloud) may serve as a more realistic reference point in large-scale adoption. Assessing BlueEdge in these hybrid environments is a critical future direction of work, as it would enable the quantification of trade-offs among latency, accuracy, energy consumption, and privacy.

## Conclusion

The current paper introduces a neural network-based solution to automated classification of data types on mobile edge devices to enhance the BlueEdge framework with smart preprocessing options. We show that, with effective architecture and feature engineering, it is possible to design a model that can properly classify data types under the stringent resource constraints of low-end mobile devices so that practical edge deployment of IoT applications can be realized. By leveraging carefully engineered features and an optimized model architecture, our technique efficiently identifies 14 distinct data types, enabling more effective preprocessing directly on the network layer.

As shown by BlueEdge, it is possible to implement practical edge intelligence using carefully designed lightweight architectures with performance comparable to cloud-based systems, and within the constraints of mobile devices. The validation in “Results” of the experiment attests to a significant reduction in the bandwidth and processing latency.

Real-world international deployment in a university registration system validated these advantages, with significant upgrades in fines, machine responsiveness, and processing speeds. Despite certain limitations in model complexity and dataset scope, our approach represents a substantial advancement in enabling intelligent information preprocessing on the community aspect, contributing to more efficient IoT-cloud communication patterns and stronger privacy protection in mobile computing environments.

Our work demonstrates the viability of deploying a state-of-the-art intelligent system on resource-limited aspect devices, proving the feasibility of edge-based preprocessing for unique data type tasks. The success of our technique suggests promising future research directions, including on-tool continual learning to conform to consumer-specific patterns, contextual classification that considers relationships among information fields, and transfer learning techniques for efficient domain adaptation.

As edge computing continues to evolve, methods like BlueEdge, which intelligently distribute preprocessing workloads between the threshold and cloud, will play an increasingly vital role in addressing the scalability, performance, and privacy demands of cutting-edge IoT ecosystems. By directing intelligence towards data sources, we will create more responsive, aid-friendly, and privacy-preserving computing environments for an increasingly interconnected world.

## Supplementary Information

Below is the link to the electronic supplementary material.

Supplementary Material 1

## Data Availability

Due to ongoing developments, the complete source code is not yet available for public viewing. Nevertheless, we will provide all the necessary details to enable anyone to replicate our findings. The initial dataset (1,400 samples) will be made available, along with the university dataset. Web link to datasets https://drive.google.com/drive/folders/1y8fPZM1MEfxIalVtzDpEzGQXZ_HaOqSa?usp=sharing. Note The dataset is free for anyone for scientific purposes, not for commercial purposes. Emails and phone numbers have been changed for personal privacy. This does not affect our experiment, which depends on the name fields for the text-matching process. the confidentiality of the dataset was ensured while preserving its ability to test and evaluate the model. When the paper is published, we plan to provide access to the trained model, code for feature extraction, and learning scripts, allowing researchers to reuse our findings and apply them in other domains. The datasets generated and analyzed during the current study are available from the corresponding author upon reasonable request. For data requests, please contact [Nagwa elmobark] at [eng_nagwaelmobark@yahoo.com].
