# Supplementary material for: BlueEdge neural network approach and its application to automated data type classification in mobile edge computing
Source: Sci Rep. 2025 Dec 12;15:43823. doi: 10.1038/s41598-025-30445-z (PMC12705763; doi:10.1038/s41598-025-30445-z)
Supplement: Supplementary file 1 — Supplementary Material 1 [file 41598_2025_30445_MOESM1_ESM.docx]

**Supplementary Material**

**BlueEdge Neural Network Approach and Its Application to Automated Data Type Classification in Mobile Edge Computing**

**Table S1: Data Type to Cleaning Strategy Mapping**

| **Data Type** | **Primary Operations** | **Matching Threshold** | **Field Type** |
| --- | --- | --- | --- |
| Person Name | Remove honorifics, normalize case, remove special chars, check nickname variations | 0.25 | text |
| Address | Normalize case, standardize abbreviations, and geocode | 0.30 | text |
| Phone Number | Remove non-digits, standardize format | 0.10 | numeric |
| Email | Lowercase, validate format | 0.00 | text |
| Date | Standardize format, validate date | 0.00 | date |
| SSN | Remove non-digits, validate format, and mask display | 0.00 | id |
| Numeric ID | Remove non-digits, validate length | 0.00 | id |
| Text Description | Normalize whitespace, spellcheck | 0.60 | text |
| URL | Lowercase, validate format, normalize protocol | 0.10 | text |
| Geographic Coordinates | Validate format, standardize format | 0.00 | numeric |
| Currency | Remove currency symbols, standardize format | 0.00 | numeric |
| Percentage | Convert to decimal, standardize format | 0.00 | numeric |
| Binary | Standardize format | 0.00 | binary |
| Categorical | Normalize case, standardize values | 0.20 | categorical |

**Table S2: Memory Usage Patterns (KB per 1000 records)**

| **Dataset Size** | **BlueEdge** | **WinPure** | **DoubleTake** | **WizSame** | **DQGlobal** |
| --- | --- | --- | --- | --- | --- |
| 1000 records | 5000 | 60000 | 60000 | 10000 | 55000 |
| 5000 records | 25000 | 300000 | 300000 | 50000 | 275000 |
| 10000 records | 50000 | 600000 | 600000 | 100000 | 550000 |

**Table S3: Evaluation Metrics Framework**

| **Performance Dimension** | **Primary Metrics** | **Measurement Method** | **Statistical Validation** |
| --- | --- | --- | --- |
| Classification Accuracy | Accuracy, Precision, Recall, F1-score | 10-fold stratified CV | McNemar's test (p<0.05) |
| Resource Consumption | Memory (bytes), CPU (%), Power (mAh) | Android Debug Bridge | Confidence intervals (95%) |
| Processing Efficiency | Time per classification, Throughput | Timestamp logging | Repeated measures (n=10) |
| Network Performance | Data reduction (%), Latency (ms) | Network monitoring | Paired t-tests |
| Robustness | Accuracy under noise, Incomplete data | Synthetic degradation | ANOVA with post-hoc |

**Table S4: Optimization Results for Mobile Edge Deployment**

| **Optimization Technique** | **Description** | **Model Size Reduction** | **Accuracy Impact** | **Notes** |
| --- | --- | --- | --- | --- |
| **8-bit Quantization** | Converted 32-bit floating-point weights to 8-bit integers for inference. | ~75% smaller model size | −0.5% (negligible) | Maintains near-identical accuracy while significantly reducing memory and latency. |
| **Pruning (Magnitude-based)** | Removed ~15% of the lowest-magnitude weights, followed by fine-tuning (10 epochs). | ~12% smaller model size | −0.8% | Improves efficiency without major accuracy degradation. |
| **Huffman Coding Compression** | Applied entropy-based weight compression after pruning. | Additional ~10% reduction | None | Lossless compression, reduces storage/transmission requirements. |
| **Combined Strategy** | Quantization + Pruning + Huffman coding. | Total reduction ~85% | < −1% overall | Best trade-off between compactness and accuracy for mobile deployment. |

**Table S5: Computational Requirements Summary**

| **Component** | **Minimum Requirements** | **Recommended** | **Optimal** |
| --- | --- | --- | --- |
| **Training** |  |  |  |
| CPU | Intel i5-8400 / AMD R5 2600 | Intel i7-10700 / AMD R7 3700X | Intel i9-11900K / AMD R9 5900X |
| RAM | 4GB | 16GB | 32GB |
| GPU | None (CPU-only) | GTX 1660 / RTX 3060 | RTX 3070+ / V100+ |
| Storage | 2GB available | 10GB SSD | 50GB NVMe SSD |
| Training Time | 4.8 hours | 2.3 hours | 45 minutes |
| **Mobile Deployment** |  |  |  |
| Android API | Level 21+ | Level 28+ | Level 30+ |
| RAM | 2GB (1GB free) | 4GB | 8GB+ |
| Storage | 100MB | 500MB | 1GB |
| Processor | ARM Cortex-A53 | ARM Cortex-A75 | Snapdragon 855+ |

**Table S6: Detailed Error Analysis for Commonly Misclassified Types**

| **Error Pattern** | **Count** | **Error Rate** | **Primary Contributing Factor** |
| --- | --- | --- | --- |
| Phone Number → Numeric ID | 4 | 4.0% | Lack of format separators |
| Numeric ID → Phone Number | 6 | 6.0% | Similar length patterns |
| SSN → Numeric ID | 5 | 5.0% | Non-standard formatting |
| SSN → Phone Number | 2 | 2.0% | Dash separator confusion |
| Date → Text Description | 3 | 3.0% | Ambiguous date formats |

**Table S7: Representative Failure Cases with Analysis**

| **Input Sample** | **True Type** | **Predicted** | **Confidence** | **Failure Reason** |
| --- | --- | --- | --- | --- |
| "1234567890" | Phone | Numeric ID | 0.82 | No separators, digits |
| "ID-123-456" | Numeric ID | Phone | 0.71 | Dash pattern similarity |
| "123456789" | SSN | Numeric ID | 0.76 | Missing XXX-XX-XXXX |
| "student_12345" | Numeric ID | Text Desc | 0.65 | Underscore as text |
| "2023/12/25" | Date | Text Desc | 0.69 | Slash format ambiguity |

**Table S8: Classification Accuracy with Incomplete Data**

| **Data Completeness** | **Neural Network** | **Rule-Based** | **RegEx Classifier** | **Cloud Sherlock** |
| --- | --- | --- | --- | --- |
| 100% (complete) | 94.2% | 87.5% | 83.9% | 92.1% |
| 80% | 91.7% | 76.3% | 68.2% | 88.4% |
| 60% | 85.3% | 59.6% | 42.1% | 79.8% |
| 40% | 72.6% | 35.2% | 21.4% | 65.3% |

**Additional Implementation Details**

**S9: Hyperparameter Optimization Results**

**Grid Search Parameters:**

- Learning rates tested: [0.0001, 0.0005, 0.001, 0.005, 0.01]
- Batch sizes tested: [16, 32, 64, 128]
- Dropout rates tested: [0.1, 0.2, 0.3, 0.4, 0.5]
- L2 regularization: [0.0001, 0.001, 0.01]

**Optimal Configuration:**

- Learning rate: 0.001
- Batch size: 32
- Dropout rate: 0.2
- L2 regularization: 0.001
- Architecture: 64-32-14 neurons

**S10: Cross-Validation Detailed Results**

**10-Fold Stratified Cross-Validation:**

- Mean accuracy: 94.2% ± 1.3%
- Standard deviation across folds: 1.3%
- Minimum fold accuracy: 92.1%
- Maximum fold accuracy: 96.8%
- Consistency metric (CV): 0.014

**S11: Feature Engineering Details**

**Complete Feature List (40 features):**

1. Total character length
2. Word count
3. Digit count
4. Alphabetic character count
5. Space count
6. Special character count 7-18. Pattern features (12 regex patterns) 19-25. Statistical features (7 ratios and entropy) 26-40. Content features (15 domain-specific indicators)
